# Cultural Adaptation and Validation of the Bangla Mental Health Literacy Scale (MHLS‐Bangla) Among Undergraduate Nursing Students

**DOI:** 10.1002/hsr2.72756

**Published:** 2026-06-30

**Authors:** Md Saleh Uddin, Towhidul Islam, Honea Ara, Ernesta Sofija, Paul Harris, S. M. Yasir Arafat

**Affiliations:** ^1^ Department of Psychiatry Bangladesh Medical University Dhaka Bangladesh; ^2^ Department of Psychiatry Sir Salimullah Medical College Mitford Hospital Dhaka Bangladesh; ^3^ Department of Psychiatry Ad‐din Women's Medical College Dhaka Bangladesh; ^4^ School of Medicine and Dentistry (Public Health) Griffith University Gold Coast QLD Australia; ^5^ School of Allied Health, Sport and Social Work Griffith University Logan QLD Australia; ^6^ Department of Psychiatry Bangladesh Specialised Hospital Dhaka Bangladesh

**Keywords:** awareness, Bangladesh, health literacy, mental health, psychometrics

## Abstract

**Background and Aims:**

Mental health literacy (MHL) is a key determinant of help‐seeking behavior and the utilization of mental health services. Currently, no validated instrument is available in Bangladesh to assess MHL. This study aimed to culturally adapt and evaluate the psychometric properties of the Bangla version of the Mental Health Literacy Scale (MHLS‐Bangla).

**Methods:**

Three tertiary care hospitals were purposively selected, and nursing students were randomly recruited. Data were collected through face‐to‐face administration in classroom settings (*N* = 224). The MHLS was adapted using a standardized process including translation, synthesis, back‐translation, expert review, and pretesting.

**Results:**

The MHLS‐Bangla comprises 35 items reflecting key domains of MHL: mental illness and treatment, mental health promotion, help‐seeking, stigma and attitude. Exploratory factor analysis using Principal Axis Factoring supported a four‐factor structure with acceptable sampling adequacy (KMO = 0.67; Bartlett's test *p* < 0.001), explaining 19.1% of the total variance. Most items loaded ≥ 0.30 on a single factor, with a small number showing weaker or cross‐loadings. Internal consistency for the total scale was acceptable (*α* = 0.75; *ω* = 0.75). The confirmatory factor analysis (CFA) was not conducted due to sample size limitations. The mean MHL score was 19.78 (SD = 4.82) out of 35.

**Conclusion:**

The MHLS‐Bangla provides a culturally adapted instrument with preliminary evidence of reliability and construct validity for assessing MHL in Bangladesh. Further validation in larger and more diverse populations is recommended to strengthen its psychometric robustness and broader applicability.

## 1 Introduction

Mental health literacy (MHL) refers to knowledge and beliefs that facilitate the recognition, management, and prevention of mental disorders, including understanding how to maintain positive mental health, recognize mental disorders and their treatments, reduce stigma, and enhance help‐seeking behaviors [1, 2]. Higher MHL enables individuals to identify mental health problems early and access appropriate support, contributing to improved outcomes at both individual and community levels. Consequently, promoting MHL has become a key focus of mental health strategies worldwide.

In Bangladesh, 6.5%–31.0% of the adult population experiences mental illness, yet 90% face a treatment gap, compounded by low awareness and high levels of stigma [3, 4, 5]. Cultural factors, including strong family involvement in health‐related decision‐making, religious and spiritual interpretations of psychological distress, and a preference for somatic explanations of emotional problems, shape perceptions of and responses to mental health issues [6, 7, 8, 9, 10]. Consequently, help‐seeking often favors family members, religious leaders, or traditional healers over formal mental health services [11]. These contextual factors underscore the importance of culturally adapting MHL measures to ensure conceptual relevance, linguistic clarity, and cultural validity within the Bangladeshi context.

Assessment of MHL varies across settings, with tools measuring domains such as knowledge, attitudes, stigma, and help‐seeking. Systematic reviews highlight the Mental Health Literacy Scale (MHLS) as a robust instrument for healthcare professionals, capturing multiple dimensions in a single tool [12, 13]. In Bangladesh, research has relied on domain‐specific scales: the 20‐item D‐Lit Bangla focuses on depression knowledge; LOSS‐Bangla (12 items) assesses suicide literacy; and SOSS‐Bangla (16 items) evaluates suicide‐related stigma across stigmatization, isolation/depression, and normalization/glorification [14, 15]. Unlike these targeted instruments, the MHLS‐Bangla (35 items) provides a broader assessment of MHL, encompassing recognition of mental disorders, help‐seeking knowledge, mental health promotion, stigma, and attitudes towards people with mental illness.

Evidence suggests that even future healthcare providers exhibit gaps in MHL. Undergraduate medical students demonstrate high stigma comparable to the general community [16], and nurses demonstrate limited recognition of psychotic symptoms despite average depression literacy [17]. Given their critical role in mental health promotion and care delivery [18], undergraduate nursing students are well positioned to support early identification, referral, and promotion of mental health. Yet, evidence on the adequacy of their MHL in Bangladesh is scarce, highlighting the need for systematic assessment.

Considering the high burden of mental disorders, limited resources, and insufficient mental health training within academic institutions, a culturally adapted and psychometrically sound MHL tool is essential. The MHLS, with its multidimensional structure and established psychometric properties, provides a suitable framework. Therefore, this study aims to culturally adapt and validate the MHLS into Bangla for undergraduate nursing students in Bangladesh.

## 2 Materials and Methods

### 2.1 Study Setting and Data Collection Procedure

Data were collected from three tertiary care hospitals, purposively selected to represent government, autonomous, and the private sectors. Three hospitals were purposively selected and they are: Sir Salimullah Medical College Mitford Hospital, Bangladesh Medical University (BMU) Hospital and Ad‐Din Women's Medical College. Data collection occurred between August 2019 and July 2020. The researcher obtained permission from the head of each nursing department, then contacted faculty coordinators responsible for students' academic activities. Invitation letters, detailing study objectives, methods, expected outcomes, and ethical considerations, were distributed to students through the coordinators. Face‐to‐face, classroom‐based administration was chosen to enhance comprehension, engagement, and response accuracy, while enabling standardized clarification of instructions. This approach also minimized non‐response bias and improved data quality.

### 2.2 Sample Estimation

Following Costello and Osborne, a minimum of five participants per item is recommended for exploratory factor analysis (EFA) [19]. With 35 items in the MHLS, the minimum required sample was 175. To strengthen statistical robustness, 300 students were randomly invited; 224 consented (74.6% response rate) and provided usable data. While sufficient for EFA, the sample size was relatively modest for confirmatory factor analysis (CFA), which is acknowledged as a limitation.

### 2.3 Instruments

#### 2.3.1 Sociodemographic Measures

A questionnaire was used to collect sociodemographic characteristics and relevant variables, including age, sex, family income, academic year, prior mental health training, and family history of mental illness. Variable selection was informed by literature identifying predictors of MHL [1, 20, 21, 22, 23].

#### 2.3.2 MHLS

The original version of the MHLS demonstrated good internal consistency and test–retest reliability [24]. This scale comprises 35 items, including the ability to recognize illness (8 items), knowledge of where to seek information (4 items), knowledge of risk factors and causes (2 items), knowledge of self‐treatment (2 items), knowledge of professional help available (3 items), and attitudes that promote recognition or appropriate help‐seeking behavior (16 items). Items 1–15 use a 4‐point likelihood scale, items 16–35 use a 5‐point agreement scale. Total score ranges from 35 to 160.

### 2.4 Adaptation of MHLS Into Bangla (MHLS Bangla)

Cross‐cultural adaptation followed established guidelines and included forward translation, synthesis, back‐translation, expert review, and pre‐testing [25, 26, 27]. Two independent forward translations (by a bilingual mental health professional and a layperson) were synthesized, followed by back‐translation by two independent bilingual translators blinded to the original instrument. An English‐language expert reviewed the reconciled version for linguistic precision and contextual relevance. The research team ensured semantic, idiomatic, experiential, and conceptual equivalence prior to committee evaluation.

The expert committee, comprising mental health professionals and bilingual experts, reviewed content validity, cultural relevance, and operational equivalence. Contextual modifications included expanding the recognition domain from 8 to 12 items to reflect locally prevalent conditions and age‐specific presentations [28]. Two items on professional confidentiality were removed, and two items on self‐treatment and mental health promotion were expanded to four items, based on local treatment gaps and stigma [28, 29]. Newly developed items were informed by literature, expert consultation, and epidemiological evidence, and were pilot tested for clarity and feasibility. Items on stigma and attitudes were retained to preserve theoretical alignment.

The final Bangla MHLS comprises 35 items across five theoretically defined domains: recognition of disorders and treatment (items 1–14), mental health promotion (15–18), knowledge of help‐seeking (19–23), stigma (24–31), and attitudes (32–35). Response options were standardized to a three‐category format: “correct/helpful/agree”, “incorrect/not helpful/disagree”, and “don't know”. For scoring, responses were dichotomized, with correct responses coded as 1 and non‐correct responses coded as 0. Pre‐final items were evaluated through expert review and a 90‐min focus group with 12 participants to assess clarity, cultural relevance, response interpretation, and overall format and layout [26, 30].

### 2.5 Data Analysis

Data were analyzed using IBM SPSS Statistics version 23 and R version 4.5.3. Internal consistency was assessed using Cronbach's alpha and McDonald's omega with values of approximately 0.70 generally considered acceptable for group‐level research [31]. Construct validity was examined using EFA with Principal Axis Factoring and direct Oblimin rotation, assuming correlated factors. As all items were dichotomously scored (correct vs. non‐correct), inter‐item associations were analyzed using phi coefficients, which are appropriate for observed binary data. Data suitability was confirmed with the Kaiser–Meyer–Olkin (KMO) measure and Bartlett's Test of Sphericity. Factors with eigenvalues > 1 were extracted, and item loadings ≥ 0.30 were considered meaningful [32]. In addition to statistical criteria, item retention was also guided by the priori coverage of the MHLS domains (mental illness and treatment, mental health promotion, help‐seeking, stigma and attitude) to ensure adequate representation of all conceptual domains during scale adaptation. Factor solutions were interpreted with reference to the original MHLS framework. CFA, differential item functioning (DIF) or item response theory (IRT) analyses were not conducted due to the sample size constraints. The dichotomous scoring format limited direct comparison with the original MHLS. These methodological considerations are acknowledged as limitations. Future studies with larger and more diverse samples and alternative scoring formats could incorporate CFA, DIF, and future evaluation of measurement equivalence [33]. Group comparisons used independent t‐tests and one‐way analysis of variance, with effect sizes reported as Cohen's *d* and *η* ^2^, respectively. All tests were two‐sided (*p* < 0.05). Prespecified analyses included sex and prior mental health training; other subgroup comparisons (academic year, religion, and family history of mental illness) were exploratory.

### 2.6 Ethical Aspects

Permission to use the original version of MHLS was obtained from Dr Matt O'Connor. The research proposal was approved by the ethical committee of *Bangladesh Medical University (BMU)*, registration number 250/6‐8‐2019. Informed consent was obtained from all participants prior to data collection, and responses were collected anonymously.

## 3 Results

### 3.1 Sample Characteristics

Among the 224 participants, the majority were female (92.9%) and enrolled in a Bachelor of Nursing program (65.9%). The mean age was 19.4 years (SD = 1.0; range 17–23 years). Most participants were Muslim (84.8%), unmarried (99.6%), had no prior mental health training (98.2%), and reported no family history of mental disorders (95.1%) (Table 1).

**Table 1:** Distribution of total MHLS Bangla Scores according to sociodemographic variables (*N* = 224).

Variable	*n* (%)	Mean (SD)
Gender		
Male	16 (7.1)	18.0 (5.7)
Female	208 (92.9)	19.7 (4.7)
Religion		
Islam	190 (84.8)	19.4 (4.8)
Hinduism	26 (11.6)	21.0 (4.7)
Christianity	7 (3.1)	20.9 (5.6)
Buddhism	1 (0.4)	21.0 (–)
Education		
BSc 1st year	69 (30.8)	18.8 (4.5)
BSc 2nd year	55 (24.6)	20.6 (5.2)
BSc 3rd year	23 (10.3)	18.9 (4.8)
Diploma 1st year	77 (34.4)	20.4 (4.8)
Prior mental health training		
Yes	4 (1.8)	23.5 (2.8)
No	220 (98.2)	19.6 (4.8)
Family history of mental illness		
Yes	11 (4.9)	20.1 (3.7)
No	213 (95.1)	19.6 (4.9)
Total	224 (100)	19.7 (4.8)

*Note:* Mean scores reflect total scores of the MHLS Bangla. Percentages and SDs are rounded to one decimal place.

Abbreviations: BSc, Bachelor of Sciences; MHLS Bangla, Bangla version of the Mental Health Literacy Scale; *n*, number of participants; %, percentage of total sample (*N* = 224); SD, standard deviation.

### 3.2 Literacy Score

The overall mean MHLS‐Bangla score indicated a moderate level of MHL within the sample (M = 19.78, SD = 4.82, Supporting Information: Table S1). No statistically significant differences were observed across sociodemographic or academic variables (all *p* > 0.05) (Supporting Information: Tables S4–S5). Item‐level performance showed higher correct response for knowledge about illness, mental health‐promoting measures and help‐seeking measures, whereas lower performance was observed for stigma and attitude related items (Supporting Information: Table S1–S3).

### 3.3 Face and Content Validity

Face and content validity were established during the cross‐cultural adaptation process through expert panel review, following established guidelines for scale adaptation and validation [25, 26].

### 3.4 Descriptive Item Analysis

Descriptive analysis of the 35‐item MHLS‐Bangla (Supporting Information: Table S1) showed substantial variability in item‐level correct response rates, ranging from 9.4% to 95.1% (M = 0.094–0.951; SD = 0.217–0.501). Several items demonstrated high correct response rates, particularly within knowledge, promotion, and help‐seeking domains, with items Q6, Q11, Q15–Q18, Q23, Q28, and Q29 showing high endorsement (≥ 0.839), suggesting potential ceiling effects. In contrast, lower correct response rates were observed for items Q3, Q4, Q24, Q33, and Q34 (≤ 0.241), indicating relatively limited correct responses for these items. Attitude‐related items (Q32–Q35) generally showed lower correct response rates (0.094–0.415), with particularly low performance on Q33 and Q34. Overall, item responses demonstrated considerable heterogeneity across domains.

### 3.5 Construct Validity

#### 3.5.1 Sampling Adequacy

The Kaiser–Meyer–Olkin (KMO) measure indicated moderate sampling adequacy (KMO = 0.67), and Bartlett's Test of Sphericity was significant Bartlett's Test of Sphericity was significant, (*χ* ^2^ (595) = 1204.42, *p* < 0.001), supporting the factorability of the correlation matrix.

#### 3.5.2 Communalities

Communalities were examined to assess the proportion of variance in each item explained by the extracted factor solution. Item communalities (*h* ^2^) ranged from 0.03 to 0.43, indicating low to moderate shared variance across items (Table 3). Variability was observed across items, with some showing relatively higher representation (e.g., Q21, Q34, and Q35) and others lower values (e.g., Q8, Q14, Q15, Q18, and Q26), reflecting differences in item‐level contribution to the factor structure.

#### 3.5.3 EFA

EFA using Principal Axis Factoring with oblique (direct Oblimin) rotation initially suggested a 5‐factor solution; however, the fifth factor was weakly defined and lacked interpretability. A 4‐factor solution was therefore retained. The final model (eigenvalues > 1) explained 19.1% of the total variance (Table 2).

**Table 2:** Total variance explained from principal axis factoring (4‐factor solution) for MHLS‐Bangla (*N* = 224).

Factor	SS loadings	Proportion of variance	Cumulative variance (%)
1	2.16	6.17	6.17
2	1.71	4.89	11.06
3	1.45	4.54	15.60
4	1.34	3.49	19.09

*Note:* Principal axis factoring with oblimin rotation was used. Factors were retained based on eigenvalues > 1 and interpretability of the factor structure. The 4‐factor solution accounted for 19.1% of the total variance.

Abbreviation: MHLS Bangla, Bangla version of the Mental Health Literacy Scale.

Most items showed primary loadings ≥ 0.30 on a single factor, yielding a generally coherent, though modest, factor structure. A small number of items showed weak loadings (e.g., Q8), cross‐loading (Q19), or a negative loading (Q24). The 4‐factor solution was retained based on statistical adequacy and interpretability criteria (Table 3).

**Table 3:** Exploratory factor analysis (principal axis factoring with Oblimin rotation) of MHLS‐Bangla (*N* = 224).

Item	Factor 1	Factor 2	Factor 3	Factor 4	*h* ^2^
Q1	0.34				0.20
Q2	0.49				0.25
Q3	0.35				0.12
Q4	0.37				0.17
Q5	0.50				0.30
Q6		0.34			0.12
Q7	0.38				0.18
Q8					0.09
Q9	0.52				0.29
Q10	0.53				0.31
Q11		0.30			0.10
Q12	0.47				0.22
Q13					0.16
Q14					0.09
Q15					0.04
Q16					0.12
Q17		0.40			0.15
Q18					0.03
Q19	0.31			0.34	0.33
Q20					0.11
Q21				0.60	0.38
Q22				0.46	0.28
Q23					0.17
Q24				−0.44	0.26
Q25		0.37			0.14
Q26					0.04
Q27					0.11
Q28			0.33		0.21
Q29		0.32			0.25
Q30		0.35			0.14
Q31		0.51			0.35
Q32			0.30		0.18
Q33			0.32		0.10
Q34			0.59		0.38
Q35			0.65		0.43

*Note:* Exploratory factor analysis was conducted using principal axis factoring with oblimin rotation (*N* = 224). A 4‐factor solution was retained based on parallel analysis, scree plot inspection, and theoretical interpretability consistent with the Mental Health Literacy framework. Only factor loadings ≥ 0.30 are presented. Communalities (*h* ^2^) represent the proportion of variance explained by the extracted solution. Loadings below 0.30 are not shown.

Abbreviation: MHLS Bangla, Bangla version of the Mental Health Literacy Scale.

#### 3.5.4 Factor Structure and Labels

The four factors were labeled based on item content, thematic coherence, and theoretical framework of MHL. The first factor (knowledge of illness and treatment) included items assessing recognition and understanding of mental health conditions (e.g., anxiety, depression, stress, schizophrenia, and obsessive–compulsive disorder). The second factor (mental health promotion) included items related to protective and healthy coping behaviors, such as sleep, exercise, emotional expression, and prayer. The third factor (help seeking and Information sources) reflected awareness and confidence in accessing mental health information and services. The fourth factor (stigma and attitudes) captured perceptions and attitudes towards mental illness and person experienced illness. This labeling supports both empirical derivation and conceptual alignment with the MHLS framework.

### 3.6 Internal Consistency

Internal consistency of the MHLS‐Bangla was assessed using Cronbach's alpha (*α*) and McDonald's omega (*ω*). The total scale demonstrated acceptable reliability (*α* = 0.75; *ω* = 0.75). At the subscale level, reliability varied across factors. The Illness and treatment subscale showed acceptable consistency, while the Mental health promotion, Help‐Seeking and information sources, and Stigma and Attitudes subscales showed lower reliability. Corrected item–total correlations ranged from 0.013 to 0.442. Overall, these findings indicate stronger reliability for the total scale than for individual subscales (Table 4).

**Table 4:** Internal consistency reliability of the MHLS‐Bangla.

Subscales	No. of items	Cronbach's *α*	McDonald's *ω*	Corrected item–total correlation range
Illness and treatment	9	0.70	0.70	0.33–0.61
Promotion	7	0.52	0.53	0.27–0.53
Help seeking	5	0.55	0.57	0.48–0.70
Stigma & attitudes	5	0.56	0.57	0.28–0.64
Total MHLS‐Bangla	35	0.75	0.75	0.013–0.442

*Note:* Corrected item–total correlations represent the correlation between each item and the total score excluding that item. Subscale reliability estimates were calculated for factor‐aligned item groupings, whereas the total scale reliability was based on all 35 retained items.

Abbreviations: *α*, Cronbach's alpha; *ω*, McDonald's omega (total); MHLS‐Bangla, Mental Health Literacy Scale–Bangla.

## 4 Discussion

### 4.1 Descriptive Analysis

The descriptive findings indicate moderate overall performance on the MHLS‐Bangla, with variability across domains. Item‐level analysis showed comparatively higher correct response rates for knowledge of mental illness, mental health promotion, and help‐seeking domains, whereas lower performance was observed for stigma and attitude‐related items. This pattern suggests that while participants demonstrate relatively stronger awareness of mental health conditions and supportive practices, attitudinal aspects and stigma‐related perceptions remain less favorable. Given the adapted response format and scoring approach, emphasis was placed on evaluating item‐level performance and distributional patterns rather than direct comparison with the original MHLS. This approach allows for a contextually appropriate interpretation of MHL within the study population while maintaining alignment with cross‐cultural validation principles.

### 4.2 Construct Validity

The EFA provided preliminary support for the construct validity of the MHLS‐Bangla. A 4‐factor solution was retained after exclusion of a weak and non‐interpretable fifth factor, explaining a modest proportion of variance [19]. The resulting structure demonstrated a coherent, though modest, multidimensional pattern, with most items loading ≥ 0.30 on a single factor. A small number of items showed weaker loadings or cross‐loadings (e.g., Q8 and Q19) or a negative loading (Q24), indicating variability in item performance. Despite this, the overall factor solution remained interpretable and broadly aligned with key domains of MHL, including illness and treatment, mental health promotion, help‐seeking, and stigma and attitudes. All items were retained to ensure adequate representation of the construct domains during the adaptation process. This reflects the balance between empirical findings and conceptual coverage commonly adopted in early‐stage psychometric validation [34, 35].

### 4.3 Internal Consistency

The MHLS‐Bangla demonstrated acceptable internal consistency at the total scale level, with Cronbach's alpha and McDonald's omega indicating adequate reliability for group‐level research (*α* = 0.75; *ω* = 0.75) [31]. This supports the overall coherence of the instrument as a multidimensional measure of MHL. Subscale reliability estimates were lower and more variable, which is not unexpected given the multidimensional structure of the scale and the relatively small number of items within some domains. This pattern likely reflects the conceptual breadth and heterogeneity of the underlying constructs rather than solely measurement limitation. While some items demonstrated weaker associations with the overall scale, their retention was considered important to preserve conceptual coverage during the adaptation process [36]. Overall, these findings suggest that the MHLS‐Bangla is suitable for exploratory and group‐level use, although certain subdomains may benefit from further refinement in future studies. Qualitative approaches, such as cognitive interviews or participant feedback, may help improve item clarity and enhance measurement precision while maintaining cultural relevance [26, 30, 37]. The reliability estimates observed in the Bangla version are comparable to those reported in other cultural adaptations of the MHLS, where moderate variability across subscales is commonly observed [24, 38, 39, 40, 41, 42, 43, 44, 45, 46, 47, 48, 49, 50, 51, 52, 53]. This pattern is consistent with the multidimensional nature of the instrument, which captures distinct yet related domains of MHL, and supports its applicability across different cultural contexts.

### 4.4 MHL of Participants

The mean MHL score among nursing students was 19.78, with no significant differences observed across sociodemographic and relevant variables (Supporting Information: Tables S4–S5). Previous studies have reported varying levels of MHL among nursing students across different settings. For example, an Indian study reported satisfactory levels of MHL [54], whereas a recent multinational study identified higher stigma levels in several Asian and Middle Eastern contexts, including India, Taiwan, Jordan, and Saudi Arabia [55], highlighting the potential influence of cultural and contextual factors. Evidence from Australia also indicates variability in MHL among nursing students despite differing socioeconomic contexts [56]. Additionally, prior exposure to individuals with mental illness has been associated with more positive attitudes, as reported in Jordan [57]. Although direct comparisons with the original MHLS are not feasible due to differences in scoring and adaptation, the observed mean score, together with variability in item‐level correct response rates (Supporting Information: Tables S1–S2), suggests heterogeneity in MHL within the sample, particularly across different domains.

### 4.5 Implications of the Study

This study provides an adapted and preliminarily validated instrument for assessing MHL in a Bangla‐speaking context. The MHLS‐Bangla can be used to evaluate MHL among nursing students and may support future research across diverse educational and community populations in Bangladesh and other Bangla‐speaking settings.

From a practical perspective, the instrument offers a structured approach for identifying domain‐specific gaps in MHL, particularly in areas such as stigma and attitudes. This may inform the development and evaluation of targeted mental health promotion, prevention, and early intervention initiatives. At a broader level, the MHLS‐Bangla has potential utility for monitoring MHL trends and supporting evidence‐informed policy and program planning.

### 4.6 Strengths and Limitations of the Study

This study is the first to adapt and validate a multidimensional MHL instrument for Bangla‐speaking populations, using a systematic process involving translation, expert review, and pilot testing to ensure conceptual and cultural appropriateness. The structured adaptation approach strengthens methodological rigor and supports the use of the instrument in a new context. Conducting the study among nursing students provides relevant insights for educational and early clinical settings, and anonymous data collection may have reduced social desirability bias.

Several limitations should be acknowledged. Test–retest reliability and criterion validity were not assessed, and CFA and DIF analyses were not conducted due to sample size constraints. The sample was limited to a single professional group, which may affect generalizability. The dichotomous scoring approach reduced response variability and limited differentiation between response types. Some items showed weaker psychometric performance (e.g., low item–total correlations, or cross‐loadings), reflecting variability in item functioning within the adapted structure.

Despite these limitations, the findings provide preliminary evidence supporting the structural validity and reliability of the MHLS‐Bangla. Future research should examine longitudinal stability, apply confirmatory methods (e.g., CFA and DIF), include more diverse populations, and explore alternative scoring approaches to enhance measurement precision and generalizability.

## 5 Conclusion

This study adapted the MHLS for use in a Bangla‐speaking context, addressing the need for a culturally appropriate tool to assess MHL in Bangladesh. The findings provide preliminary evidence supporting the reliability and construct validity of the MHLS‐Bangla and highlight its potential utility for research and educational applications. Further validation in larger and more diverse populations is recommended to strengthen its psychometric properties and broader applicability.

## Author Contributions

**Md Saleh Uddin:** Conceptualization, data curation, formal analysis, funding acquisition, writing – original draft, methodology, investigation, project administration, visualization. **Towhidul Islam:** Conceptualization, methodology, investigation. **Honea Ara:** Conceptualization, methodology, investigation. **Ernesta Sofija:** Supervision, writing – review and editing, formal analysis, methodology. **Paul Harris:** Writing – review and editing, supervision, methodology, formal analysis. **S. M. Yasir Arafat:** Conceptualization, methodology, supervision, writing – review and editing, investigation, formal analysis.

## Disclosure

ChatGPT (OpenAI; GPT‐5.2) was used solely to assist with language editing and grammatical refinement to improve the clarity and readability of the manuscript. The tool was not used for content generation, data analysis, interpretation, or decision‐making. All intellectual content and responsibility for the manuscript remain with the authors.

## Ethics Statement

Permission to use the original version of MHLS was obtained from Dr Matt O'Connor. The research proposal was approved by the ethical committee of *Bangladesh Medical University (BMU)*, registration number 250/6‐8‐2019. Informed consent was obtained from all participants prior to data collection, and responses were collected anonymously.

## Conflicts of Interest

S.M. Yasir Arafat is an Editorial Board member of Health Science Reports and author of this article. To minimize bias, he was excluded from all editorial decision‐making related to the acceptance of this article for publication.

## Transparency Statement

The lead author Md Saleh Uddin affirms that this manuscript is an honest, accurate, and transparent account of the study being reported; that no important aspects of the study have been omitted; and that any discrepancies from the study as planned (and, if relevant, registered) have been explained.

## Supporting information

Supporting File

## Data Availability

The data that support the findings of this study are available on request from the corresponding author.
