# Supplementary material for: Cultural Adaptation and Validation of the Bangla Mental Health Literacy Scale (MHLS‐Bangla) Among Undergraduate Nursing Students
Source: Health Sci Rep. 2026 Jun 30;9(7):e72756. doi: 10.1002/hsr2.72756 (PMC13316816; doi:10.1002/hsr2.72756)
Supplement: Supplementary file 1 — Supporting File [file HSR2-9-e72756-s001.docx]

**Supplementary material**

**Table S1**
*Descriptive Statistics for All Items and Total Score of the Mental Health Literacy Scale Bangla Version (MHLS-Bangla, N = 224)*

| **Item** | **M** | **SD** |
| --- | --- | --- |
| Q1 | 0.518 | 0.501 |
| Q2 | 0.473 | 0.500 |
| Q3 | 0.201 | 0.402 |
| Q4 | 0.232 | 0.423 |
| Q5 | 0.295 | 0.457 |
| Q6 | 0.915 | 0.279 |
| Q7 | 0.643 | 0.480 |
| Q8 | 0.594 | 0.492 |
| Q9 | 0.344 | 0.476 |
| Q10 | 0.272 | 0.446 |
| Q11 | 0.880 | 0.326 |
| Q12 | 0.460 | 0.500 |
| Q13 | 0.772 | 0.420 |
| Q14 | 0.442 | 0.498 |
| Q15 | 0.920 | 0.272 |
| Q16 | 0.839 | 0.368 |
| Q17 | 0.938 | 0.243 |
| Q18 | 0.951 | 0.217 |
| Q19 | 0.554 | 0.498 |
| Q20 | 0.603 | 0.490 |
| Q21 | 0.772 | 0.420 |
| Q22 | 0.746 | 0.437 |
| Q23 | 0.853 | 0.355 |
| Q24 | 0.241 | 0.429 |
| Q25 | 0.429 | 0.496 |
| Q26 | 0.446 | 0.498 |
| Q27 | 0.335 | 0.473 |
| Q28 | 0.906 | 0.292 |
| Q29 | 0.848 | 0.360 |
| Q30 | 0.563 | 0.497 |
| Q31 | 0.750 | 0.434 |
| Q32 | 0.380 | 0.486 |
| Q33 | 0.094 | 0.292 |
| Q34 | 0.161 | 0.368 |
| Q35 | 0.415 | 0.494 |
| **Total** | 19.781 | 4.820 |

***Note:*** N = 224. Mean, standard deviation (SD), skewness, and kurtosis are reported for each item and the total score of the MHLS Bangla. Skewness and kurtosis indicate the shape of the item distributions; extreme values reflect items with very high or very low proportions of correct responses.

**Table S2**

*Distribution of Correct Responses for Each Item of the Mental Health Literacy Scale Bangla Version (MHLS-Bangla, N = 224)*

| **Item** | **Item Description** | **n** | **Correct response (%)** |
| --- | --- | --- | --- |
| 1 | Major Depressive Disorder | 224 | 51.8 |
| 2 | Anxiety Disorder | 224 | 47.3 |
| 3 | Obsessive Compulsive Disorder | 224 | 20.1 |
| 4 | Conversion Disorder | 224 | 23.2 |
| 5 | Bipolar Mood Disorder | 224 | 29.5 |
| 6 | Substance Use Disorder | 224 | 91.5 |
| 7 | Sexual Dysfunction | 224 | 64.3 |
| 8 | Personality Disorder | 224 | 59.4 |
| 9 | Schizophrenia | 224 | 34.4 |
| 10 | Oppositional Defiant Disorder & Conduct Disorder | 224 | 27.2 |
| 11 | Intellectual Disability & Autism Spectrum Disorder | 224 | 87.9 |
| 12 | Dementia | 224 | 54.0 |
| 13 | Scientific treatment availability for mental illness | 224 | 77.2 |
| 14 | Effectiveness of pharmacological & psychological treatments | 224 | 44.2 |
| 15 | Benefits of sleep, food and rest during stress or anxiety | 224 | 92.0 |
| 16 | Coping during stress, anxiety or depression | 224 | 83.9 |
| 17 | Seeking social support during stress or anxiety | 224 | 93.8 |
| 18 | Expressing emotion, keeping social contact & religious practice during stress or anxiety | 224 | 95.1 |
|  |  |  |  |

***(Continue)***

**Table S2 (Continue)**

*Distribution of Correct Responses for Each Item of the Mental Health Literacy Scale Bangla Version (MHLS-Bangla, N = 224)*

| **Item** | **Item Description** | **n** | **Correct response (%)** |
| --- | --- | --- | --- |
| 19 | Confident about where to seek information on mental illness | 224 | 55.4 |
| 20 | Confident using computer or phone to seek information about mental illness | 224 | 60.3 |
| 21 | Confident attending appointment to seek information about mental illness | 224 | 77.2 |
| 22 | Confident having access to resources to seek information about mental illness | 224 | 74.6 |
| 23 | Seeking help from mental health professionals for mental health issues | 224 | 85.3 |
| 24 | People with mental illness can snap out if they want | 224 | 24.1 |
| 25 | Mental illness a sign of personal weakness | 224 | 42.9 |
| 26 | Mental illness is not a medical illness | 224 | 44.6 |
| 27 | People with mental illness are dangerous | 224 | 33.5 |
| 28 | Best to avoid people with mental illness so that you don’t develop this problem | 224 | 90.6 |
| 29 | If I had a mental illness I would not tell anyone | 224 | 84.8 |
| 30 | Seeing mental health professional means not strong enough to manage own difficulties | 224 | 56.3 |
| 31 | Believe that treatment provided by mental health professionals are not effective | 224 | 75.0 |
| 32 | Willingness to work with a colleague having mental illness | 224 | 37.9 |
| 33 | Willingness regarding someone of own family marrying a person with mental illness | 224 | 9.4 |
| 34 | Willingness to vote for politicians who had experienced mental illness | 224 | 16.1 |
| 35 | Willingness to employ someone who had experienced mental illness | 224 | 41.5 |

***Note:*** n = number of participants; % = percentage of participants providing a correct response. Items grouped into subscales: ability to recognise disorders and treatment (items 1-14), mental health promotion measures (items 15-18), knowledge of help-seeking measures (item 19-23), stigma towards mental illness (items 24-31) and attitude towards persons with mental illness (item 32-35). Higher percentages indicate better knowledge or positive attitudes in that domain.

**Table S3**

*Descriptive Statistics for the Four Subscales of the Mental Health Literacy Scale Bangla Version (MHLS-Bangla, N = 224)*

| **Variable** | **Min** | **Max** | **Mean** | **SD** |
| --- | --- | --- | --- | --- |
| Mental illness & treatment | 0.00 | 14.00 | 7.04 | 2.73 |
| Mental health promotion | 0.00 | 4.00 | 3.64 | 0.61 |
| Help-Seeking | 0.00 | 5.00 | 3.52 | 1.30 |
| Stigma and attitude | 0.00 | 11.00 | 5.56 | 2.14 |
| **Total Score** | **6.00** | **34.00** | **19.78** | **4.82** |

**Note.** Min = Minimum score, Max = Maximum score, Mean = Mean score, SD = standard deviation.

**Table S4**
*Independent Samples t-Tests for Total Score by Gender, Prior Mental Health Training, and Experience of Family Members’ Mental Illness*

| **Variable** | **Group** | **N** | **Mean ± SD** | **t** | **df** | **Mean Difference** | **95% CI of Difference** | ***p* (Two-tailed)** |
| --- | --- | --- | --- | --- | --- | --- | --- | --- |
| Gender | Male | 16 | 18.31 ± 5.76 | -1.27 | 222 | -1.58 | -4.04 to 0.88 | 0.207 |
|  | Female | 208 | 19.89 ± 4.74 |  |  |  |  |  |
| Prior MH Training | Yes | 4 | 23.50 ± 3.70 | 1.56 | 222 | 3.79 | -0.99 to 8.56 | 0.120 |
|  | No | 220 | 19.71 ± 4.82 |  |  |  |  |  |
| Family Members’ Mental Illness | Yes | 11 | 20.36 ± 3.72 | 0.41 | 222 | 0.61 | -2.33 to 3.55 | 0.682 |
|  | No | 213 | 19.75 ± 4.87 |  |  |  |  |  |

***Note:*** Values are Mean ± SD. Independent t-tests compare total scores between groups. Table reports t, df, mean difference, 95% CI, and two-tailed p. Levene’s test was not significant; equal variances assumed. Higher scores indicate higher total scores.

**Table S5**
*One-Way ANOVA Summary for Total Score by Religion and Education*

| **Variable** | **F** | **df (Between, Within)** | ***p*** | **η² (95% CI)** |
| --- | --- | --- | --- | --- |
| Religion | 0.855 | 3, 220 | 0.465 | 0.012 (0.000–0.041) |
| Education | 2.267 | 3, 219 | 0.082 | 0.031 (0.000–0.072) |

***Note:*** F = F-statistic; df = degrees of freedom; η² = eta-squared (effect size) with 95% confidence interval. Levene’s test indicated equal variances for both variables (p > 0.05). Post hoc comparisons were not conducted as omnibus ANOVA tests were not statistically significant.
